# 3p21.3 tumor suppressor gene RBM5 inhibits growth of human prostate cancer PC-3 cells through apoptosis

**DOI:** 10.1186/1477-7819-10-247

**Published:** 2012-11-16

**Authors:** Lijing Zhao, Ranwei Li, Chen Shao, Ping Li, Jian Liu, Ke Wang

**Affiliations:** 1 Department of Respiratory Medicine, The Second Affiliated Hospital of Jilin University, Changchun, Jilin, 130041, China; 2 Department of Pathophysiology, Norman Bethune College of Medicine of Jilin University, Changchun, Jilin, 130021, China; 3 Department of Urinary Surgery, The Second Affiliated Hospital of Jilin University, Changchun, Jilin, 130041, China; 4 Department of Digestive Medicine, China-Japan Union Hospital of Jilin University, Changchun, Jilin, 130033, China

**Keywords:** RBM5, Prostate cancer, Apoptosis, PC-3, Gene expression

## Abstract

**Background:**

Recent studies have indicated that the nuclear RNA-binding protein RBM5 has the ability to modulate apoptosis and suppress tumor growth. The aim of this study is to investigate the expression of RBM5 in human prostate cancer and its mechanism of tumor suppression.

**Methods:**

The expression of RBM5 protein in cancerous prostatic tissues and normal tissues was examined by IHC. PC-3 cell line was used to determine the apoptotic function of RBM5 *in vitro*. PC-3 cells were transiently transfected with pcDNA3.1-RBM5. Cell viability was determined by MTT assay. Rhodamine 123 staining and Annexin V analysis were performed to observe the apoptotic activity of PC-3 cells overexpressing RBM5. Expression of apoptosis-related genes was assessed by western blot.

**Results:**

The expression of RBM5 protein was significantly decreased in cancerous prostatic tissues compared to the normal tissues. PC-3 cells overexpressing RBM5 showed not only significant growth inhibition compared with the vector controls, but also dysfunction of mitochondrial membrane potential and increased apoptotic activity. To further define RBM5 function in apoptotic pathways, we investigated differential expression profiles of various BH3-only proteins including Bid, Bad, and Bim, and apoptosis regulatory proteins include P53, cleaved caspase9, and cleaved caspase3. We found that the expression of both BH3-only proteins and apoptosis regulatory proteins was increased in RBM5 transfected cells.

**Conclusion:**

The expression of RBM5 protein was significantly decreased in cancerous prostatic tissues, which suggests that RBM5 plays an important role in the pathogenesis of prostate cancer. RBM5 may induce the apoptosis of prostate cancer PC-3 cells by modulating the mitochondrial apoptotic pathway, and thus RBM5 might be a promising target for gene therapy on prostate cancer.

## Background

Prostate cancer (PCa) is the second most common cause of cancer and the sixth leading cause of cancer death among men worldwide with an estimated 899,000 new cases and 258,000 new deaths in 2008. The worldwide PCa burden is expected to grow to 1.7 million new cases and 499,000 new deaths by 2030 simply due to the growth and aging of the global population [1]. In China, the incidence of prostate cancer has increased over time [2]. Surgery and radiotherapy with or without androgen deprivation therapy are the most effective therapeutic strategies for localized disease [3]. However, there is currently no effective treatment strategy for patients with advanced prostate cancer at diagnosis and those who do not respond to primary curative attempts. Chemotherapeutic drugs can only extend the lives of patients with advanced prostate cancer by months, and they are also associated with dose-related toxicity and the emergence of tumor cell resistance to the drugs. It is imperative to develop novel, more effective strategies to treat prostate cancer. With the recent advances in the understanding of molecular pathways involved in prostate cancer progression, targeted therapies that are designed to interfere with the way cancer cells grow and survive offer new hope in prostate cancer therapeutics.

RNA-binding motif protein 5 (RBM5) is a gene which maps to human chromosome 3p21.3, a critical region which is deleted in a large number of human cancers and which is predicted to contain one or more tumor suppressor genes (TSGs) [4,5]. The expression level of RBM5 was shown to be high in the adult thymus and low in the fetal thymus, indicating that RBM5 expression may be developmentally regulated [6]. Previous studies have shown that RBM5 mRNA and protein expression was frequently reduced in different cancers, including ras-transformed Rat-1 embryonic fibroblastic cells [7], breast cancer [8], human vestibular schwannoma [9], and primary lung cancer specimens [10,11]. A number of lines of evidence indicate involvement of RBM5 and its splice variants in apoptosis, cell proliferation, and oncogenesis [12,13]. Introduction of RBM5 cDNA into human breast cancer cells deleted of 3p21.3 reduced both anchorage-dependent and -independent cell growth *in vitro*[10]. The ectopic expression of RBM5 suppresses the growth of human lung cancer [14,15], breast cancer [16], renal tumors [17], fibrosarcoma [8], and hematopoietic cells [7,18,19].

These observations suggest that changes in either the level of expression of RBM5, or in the processing of its mRNA, have profound consequences for cellular behavior and suggest a role for RBM5 as one of the tumor suppressor genes. Although the mechanisms of RBM5-mediated tumor suppression remain unknown, recent studies suggest that RBM5 is involved in the regulation of the apoptosis pathway [12,13]. Defects in apoptosis underpin both tumorigenesis and drug resistance [20]. However, the role of RBM5 in prostate cancer has yet to be elucidated.

Hormone-refractory prostate cancer (HRPC), resistant to hormone therapy, is a major obstacle in clinical treatment [21]. Accordingly, the approach for HRPC treatment is deeply demanded. In this study, we focused our research specifically on HRPC cell line PC-3 cells. In the preliminary work, our group had found that human lung cancer tissues expressed less RBM5 than normal tissues did [11], and overexpression of RBM5 can inhibit the proliferation and induce the apoptosis of human lung cancer A549 cells [15]. In this study, we hypothesized that RBM5 may be involved in human prostate cancer cells. Our research revealed that overexpression of RBM5 induced the apoptosis of prostate cancer PC-3 cells by promoting the mitochondrial apoptotic pathway, activating the caspase-9- and caspase-3-dependent apoptosis. These findings suggest that RBM5 may act as a prognostic indicator in prostate cancer patients, as well as act as a molecular target for gene therapy.

## Methods

### Prostate tissue acquisition and immunohistochemistry staining (IHC)

Prostate cancer specimens were obtained from patients undergoing prostatectomy in the Second and the Third Affiliated Hospitals of Jilin University, the Gleason scores for which were from grade 7 to grade 8. Normal prostate tissues were obtained from patients undergoing surgery for benign prostatic hyperplasia (BPH) in these hospitals. This study was approved by the Research Ethics Committee of Norman Bethune College of Medicine, Jilin University and was in compliance with the Helsinki Declaration. The tissues were examined by the pathologists to confirm the diagnosis before IHC analyses. All specimens were fixed in 10% formalin, embedded in paraffin, and cut into 4-μm-thick slides. The slides were dewaxed, and the endogenous peroxidase activity was blocked by treatment with 3% hydrogen peroxide solution in methanol for 20 min. Non-specific binding was prevented by blocking with normal goat serum (1:10) for 10 min. The staining procedure was carried out using an avidin-biotin-peroxidase complex method. The presence of RBM5 was evaluated by staining with rabbit anti-RBM5 antibody (Abcam, Cambridge, MA, USA). After incubation with the primary antibody for 60 min, the slides were incubated with the biotinylated goat anti-rabbit IgG (H+L) (DAKO, Carpinteria, CA, USA) at 37°C for 30 min, followed by incubation with a 1:200 streptavidin-biotin-peroxidase complex (Sigma, St. Louis, MO, USA) for 30 min. Reactive products were visualized with 3,3′-diaminobenzidene (DAB) as the chromogen, and the slides were counterstained with hematoxylin. Sections previously known to express RBM5 were included in each run, receiving either the primary antibody as the positive control, or a mouse IgG as the negative control. The stained slides were analyzed with a microscope at 400× magnification. Cellular brownish staining was scored as positive and the threshold was set at 10%.

### Cell culture and transfection

The human prostate cancer cell line PC-3 was obtained from the American Type Culture Collection (ATCC, Rockville, MD, USA) and incubated at 37°C with humidified 5% CO_2_ in IMDM (Hyclone, UT, USA) supplemented with 10% fetal bovine serum (Hyclone, UT, USA), 100 unit/mL of penicillin, 100 μg/mL of streptomycin, and 2 mM of L-glutamine. 5 × 10^5^ cells were plated onto 6-well plates 24 h before transfection. The pcDNA3.1-RBM5 and pcDNA3.1 plasmids were provided by Dr. Leslie C. Sutherland from Research Program, Northeast Cancer Centre, Health Sciences North in Canada. PC-3 cells were transfected using lipofectamine 2000 (Invitrogen, Carlsbad, CA, USA) with 4 mg DNA according to the manufacturer’s instructions. Transfection media were removed 6 h after transfection and replaced with fresh complete medium containing 10% fetal bovine serum (FBS). Controls included lipofectamine 2000 treated cells and empty vector pcDNA3.1 transfected cells.

### Semi-quantitative reverse transcription PCR (RT-PCR) and western blot analysis

PC-3 cells were divided into three groups: (a) control (mock-transfected); (b) EV (transfected with empty vector pcDNA3.1); (c) RBM5 (transfected with pcDNA3.1-RBM5), and cells were harvested after 48 h following transfection and treatment. Overexpression of RBM5 mRNA and protein was confirmed by RT-PCR and western blot.

For semi-quantitative RT-PCR, total RNA was extracted from the cells using the Trizol reagent (Invitrogen, Carlsbad, CA, USA) according to the manufacturer’s instructions. First-strand cDNA was generated by reverse transcription of 1 μg RNA samples using a SuperScript pre-amplification system (Promega, Madison, MI, USA). One-tenth of the reverse-transcribed RNA was used in the PCR reaction. The primer sequences were as follows: GAPDH sense: 5′-GAAGGTGAAGGTCGGAGTC-3′ and antisense: 5′-GAAGATGGTGATGGGATTTC-3′; RBM5 sense: 5′-GCACGACTATAGGCATGACAT-3′ and antisense: 5′-AGTCAAACTTGTCTGCTCCA-3′. The PCR products were separated by electrophoresis on 1% agarose gels. The PCR products were visualized by a Tanon-1600 figure gel image processing system and analyzed with a GIS 1D gel image system software (Tanon, Shanghai, China).

For western blot analysis, cells were lysed with HEPES lysis buffer. The cell lysates were then centrifuged for 30 min at 12,000×g and the protein concentrations were determined using the Protein Assay Kit (Bio-Rad, Hercules, CA, USA). Forty micrograms of protein were separated by 10% SDS-PAGE gel and transferred onto a PVDF membrane (Millipore, Bedford, MA, USA). The membranes were blocked with 5% non-fat milk for 1 h at room temperature and probed with antibodies of anti-RBM5 (Abcam, Cambridge, MA, USA), anti-Caspase3 (Cell Signaling Technology, Boston, MA, USA), anti-Caspase9 (Cell Signaling Technology, Beverly, MA, USA), anti-Bid, anti-Bim, anti-Bad (Cell Signaling Technology, Danvers, MA, USA), and anti-β-actin (Santa Cruz Biotechnology, Santa Cruz, CA, USA) overnight at 4°C. Horseradish peroxidase-conjugated secondary antibodies (Thermo, Waltham, MA, USA) at a dilution of 1:2,000 were then applied for 1 h at room temperature. The protein bands were then detected using an Enhanced Chemiluminescece kit (Pierce Biotechnology Ltd., Rockford, IL, USA). The protein levels were quantified by densitometry using Quantity One software (Bio-Rad).

### Cell viability assays

Cell viability was determined using 3-(4,5-dimethylthia-zol-2-yl)-2,5-diphenyltetrazolium bromide (MTT) assays. The cells were plated onto 96-well plates at a concentration of 1×10^4^ cells per well for 24 h and then transfected with pcDNA3.1 or pcDNA3.1-RBM5 for 24 h, 48 h, and 72 h. The cells were treated with 0.5 mg/mL MTT (Sigma-Aldrich, St. Louis, MO, USA) solution for 4 h. The medium was removed, and 100 mL of dimethylsulfoxide was added to each well. The formazan dye crystals were solubilized for 15 min and the optical density was measured at 570 nm with a Vmax Microplate Reader (Molecular Devices, Sunnyvale, CA, USA). Cell viability was calculated as a percentage of the control (untreated) values. Experiments were repeated three times with each experiment containing triplicate samples.

### Rhodamine123 staining for mitochondria membrane potentials

Mitochondria membrane potentials were assessed by Rhodamine123 (Sigma-Aldrich, St. Louis, MO, USA) staining. After transfected by plasmid for 48 h, PC-3 cells were incubated with 2 μM Rhodamine123 at 37°C for 30 min, washed with phosphate-buffer solution, and examined using a fluorescence microscope (Olympus, Japan) to observe the mitochondria membrane potentials.

### Flow cytometry analysis of apoptosis

After transfected by plasmid for 48 h, PC-3 cells were trypsinized and centrifuged, washed, and stained using the Annexin-V-FITC Apoptosis Detection Kit (Beyotime, Shanghai, China). The samples were then analyzed for apoptosis by a FACScan flow cytometer (Becton Dickinson, Franklin Lakes, NJ, USA). Annexin-V-FITC(−)/PI(−) was used to indicate cells that had survived, Annexin-V-FITC(+)/PI(−) was used to indicate cells that were in the early stage of apoptosis, and Annexin-V-FITC(+)/PI(+) was used to indicate cells in the late stages of apoptosis or necrosis.

### Statistics

Chi-square test was used to analyze the difference in RBM5 expression between normal and cancerous prostate tissues. Data were presented as mean ± standard deviation (SD) when appropriate. The regression analysis was performed with SPSS software (version 17.0). Comparisons between treatments were made using a paired Student’s *t*-test, or one-way ANOVA for multiple group comparisons. A *P* value of <0.05 was considered statistically significant.

## Results

### Prostate cancer tissues expressed less RBM5

**Figure 1:**
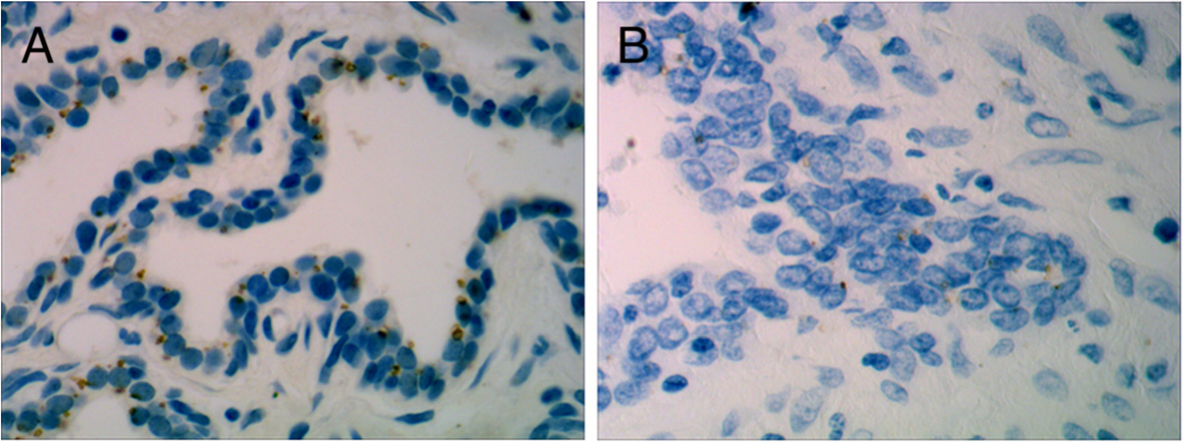
**Immunohistochemistry staining of RBM5 in normal and cancerous prostatic tissue.** (**A**) Normal prostate. (**B**) Prostate cancer. The images were obtained at 400 × magnifications, brown color represents RBM5 staining.

We first analyzed the expression of RBM5 by IHC staining in a collection of prostate cancer and normal tissues in a Chinese cohort. As shown in Figure 1, the RBM5 protein was virtually undetectable in prostate cancer but highly expressed in normal prostatic tissue. The cellular localization of RBM5 is mostly cytoplasmic. Overall, RBM5 was expressed in 10 of 11 (90.9%) normal prostate tissues, and the cytoplasmic/nuclear ratio of the staining was about 50%; while the RBM5 was expressed in two of 12 prostate cancer specimens (16.7%), and the cytoplasmic/nuclear ratio of the staining was about 10%. The difference is statistically significant (*P* <0.01). These results indicated that RBM5 is less-expressed in clinical prostate cancers, suggesting RBM5 is a promising target for prostate cancer therapy.

### RBM5 was overexpressed in pcDNA3.1-RBM5 transfected PC-3 cells

As a eukaryotic expression, pcDNA3.1 can transfer the ectopic gene into cells, and express the interest protein in the target cells. In this study, we employed PC-3 cells to investigate the function of RBM5 in prostate cancer cells.

**Figure 2:**
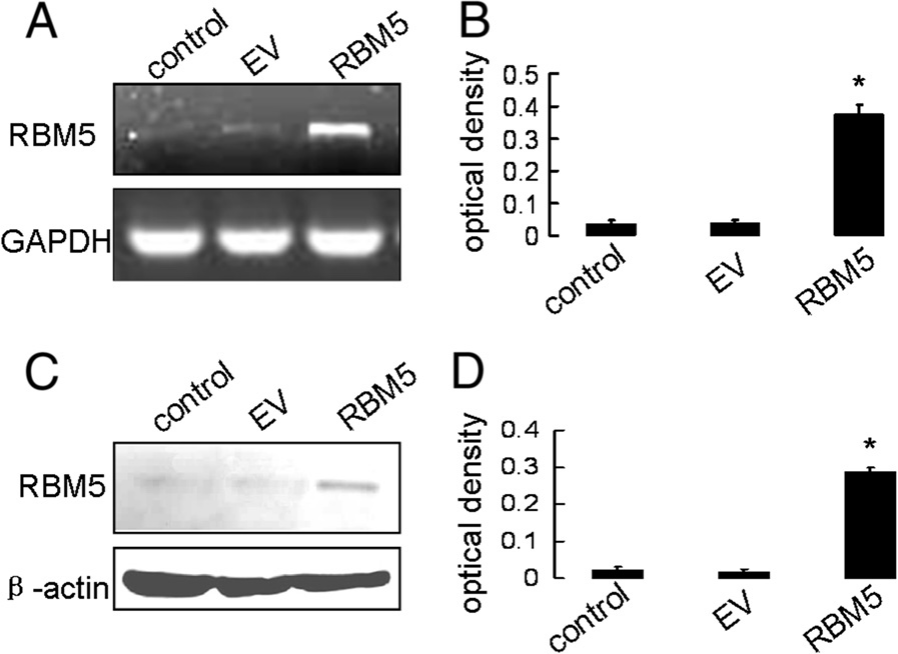
**Overexpression of RBM5 in PC-3 cells.** PC-3 cells were transfected with pcDNA3.1 or pcDNA3.1-RBM5 plasmids for 48h. RT-PCR and western blot were performed respectively to determine the RBM5 mRNA (**A**) and protein levels (**C**). Data shown are means±S.D. of three separate experiments (**C** for mRNA and **D** for protein). * indicates significant difference as compared to the mock control and EV (*P* <0.01).

After transfection with pcDNA3.1 vector and pcDNA3.1-RBM5 vector, semi-quantitative RT-PCR and western blot were performed to analyze the expression of RBM5 mRNA and protein. As shown in Figure 2A and 2B, the expression of RBM5 mRNA increased significantly in transfected pcDNA3.1-RBM5 cells compared with transfected pcDNA3.1 vector and mock control cells (treated with lipofectamine 2000 only) (*P* <0.01). Western blot assay showed a similar and statistically significant increase (*P* <0.01) (Figure 2C and 2D). The result demonstrated that RBM5 was effectively overexpressed in the pcDNA3.1-RBM5 transfected cells (RBM5), compared with the cells transfected with empty vector pcDNA3.1 (EV).

### Overexpression of RBM5 inhibited proliferation of PC-3 cells

**Figure 3:**
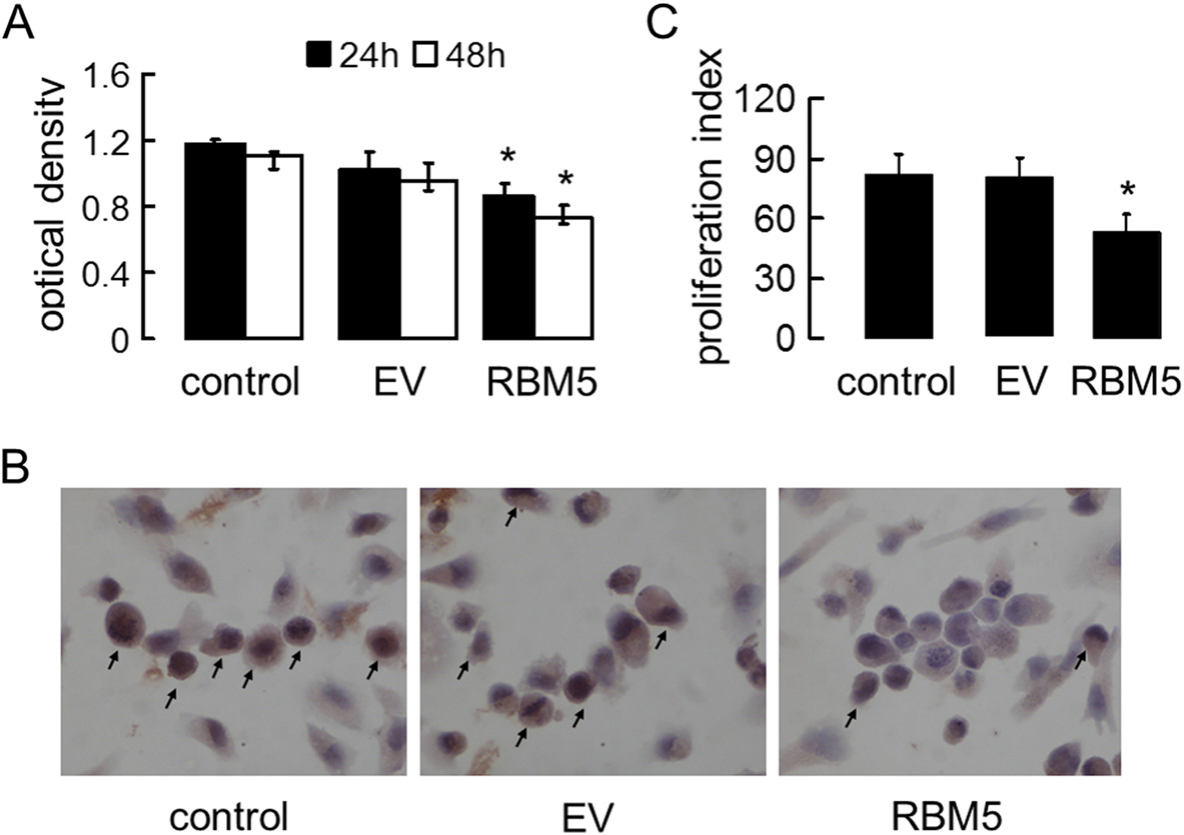
**RBM5 inhibits proliferation of PC-3 cells.** PC-3 cells were transfected with pcDNA3.1 or pcDNA3.1-RBM5 plasmids for up to 24 h and 48 h. Afterwards, (**A**) cell growth was evaluated by MTT assay. The data were presented as optical density in different groups. Data shown are means ± S.D. of three separate experiments. (**B**) Immunocytochemistry staining of PCNA. (**C**) Proliferation index of PCNA. * indicates significant difference as compared to the control and EV (*P* <0.05).

To explore the effect of RBM5 overexpression on cell growth, MTT assays were performed at 24 h, 48h, and 72 h, respectively, after the transfection. Results showed that there was a significant inhibition of cell proliferation in RBM5 overexpressing PC-3 cells compared with the mock control and EV groups (Figure 3A). Along with the extension of the time, the inhibition rate was increased, and at 72 h, the inhibition rate had been more than 30% (data not shown).

To further explore the effect of RBM5 overexpression on proliferation of PC-3 cells, Immunocytochemistry staining was employed to detect the expression of PCNA after transfected. As shown in Figure 3B, the staining results showed that the lowest numbers of proliferating cells (PCNA positive) were found in pcDNA3.1-RBM5 transfected group. Quantitative analyses of the stained slides were performed and the results are summarized in Figure 3C. The proliferation index was defined as percent of tumor cells stained positively for PCNA. In RBM5 group, the proliferation index was significantly lower (*P* <0.01), when compared with the control groups.

### Overexpression of RBM5 induced apoptosis of PC-3 cells

**Figure 4:**
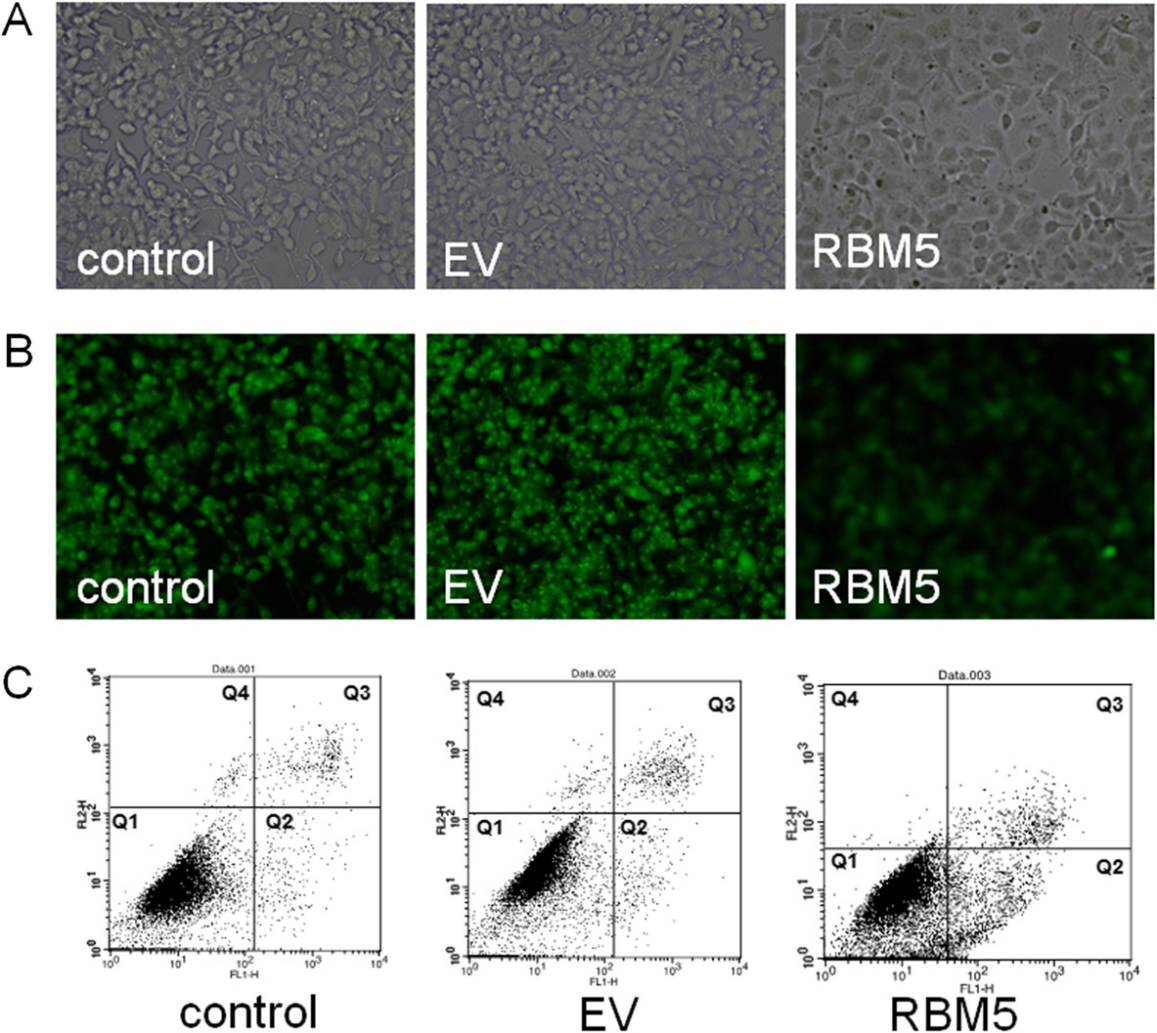
**RBM5 induces dysfunction of mitochondrial membrane potential and modulates apoptosis of PC-3 cells.** (**A**) Morphological images of PC-3 cells transfected with pcDNA3.1 and pcDNA3.1-RBM5. (**B**) Analysis of mitochondrial membrane potential by Rhodamine123 staining in PC-3 cells under the fluorescence microscope. (**C**) Flow cytometry analysis of apoptosis by Annexin V staining. The representative data of FCM with Annexin V and PI staining for detecting apoptotic cells were shown in controls and RBM5-transfected groups. Q1 represents normal cells, Q2 and Q3 represent early and late apoptotic cells, respectively, Q4 represents necrotic cells.

To determine the contribution of inhibition of cell growth induced by RBM5 overexpression, we employed Rhodamine 123 staining and flow cytometry analysis to determine apoptotic activity in PC-3 cells. Rhodamine 123 can easily be absorbed by mitochondrial membrane, the fluorescence intensity depends on mitochondrial membrane potential, can be used in analysis of apoptosis. As showed in Figure 4B, the fluorescence intensity was significantly decreased in the PC-3 cells treated with pcDNA3.1-RBM5 compared with the control groups, which suggested the dysfunction of mitochondrial membrane potential.

Flow cytometry analysis was used to detect apoptotic cells which are characterized with phosphatidyl serine (PS) translocation on the outer cell membrane. Cells were double-stained with Annexin V and PI after 48 h transfection. The early and the late apoptotic cells were distributed in the Q2 and Q3 regions, respectively. The necrotic cells were located in the Q4 region. Representative raw FCM data are as follows: cells transfected with RBM5 showed a higher proportion of early and late apoptosis (11.16%±2.53% and 1.07%±0.18%) as compared to the control cells (3.49%±1.17% and 0.81%±0.13%) and EV cells (3.55%±0.62 and 0.92%±0.15%), respectively (Figure 4C). The result suggested that overexpression of RBM5 significant increased apoptosis in PC-3 cells.

### RBM5 overexpression was associated with alteration of apoptosis-related genes

**Figure 5:**
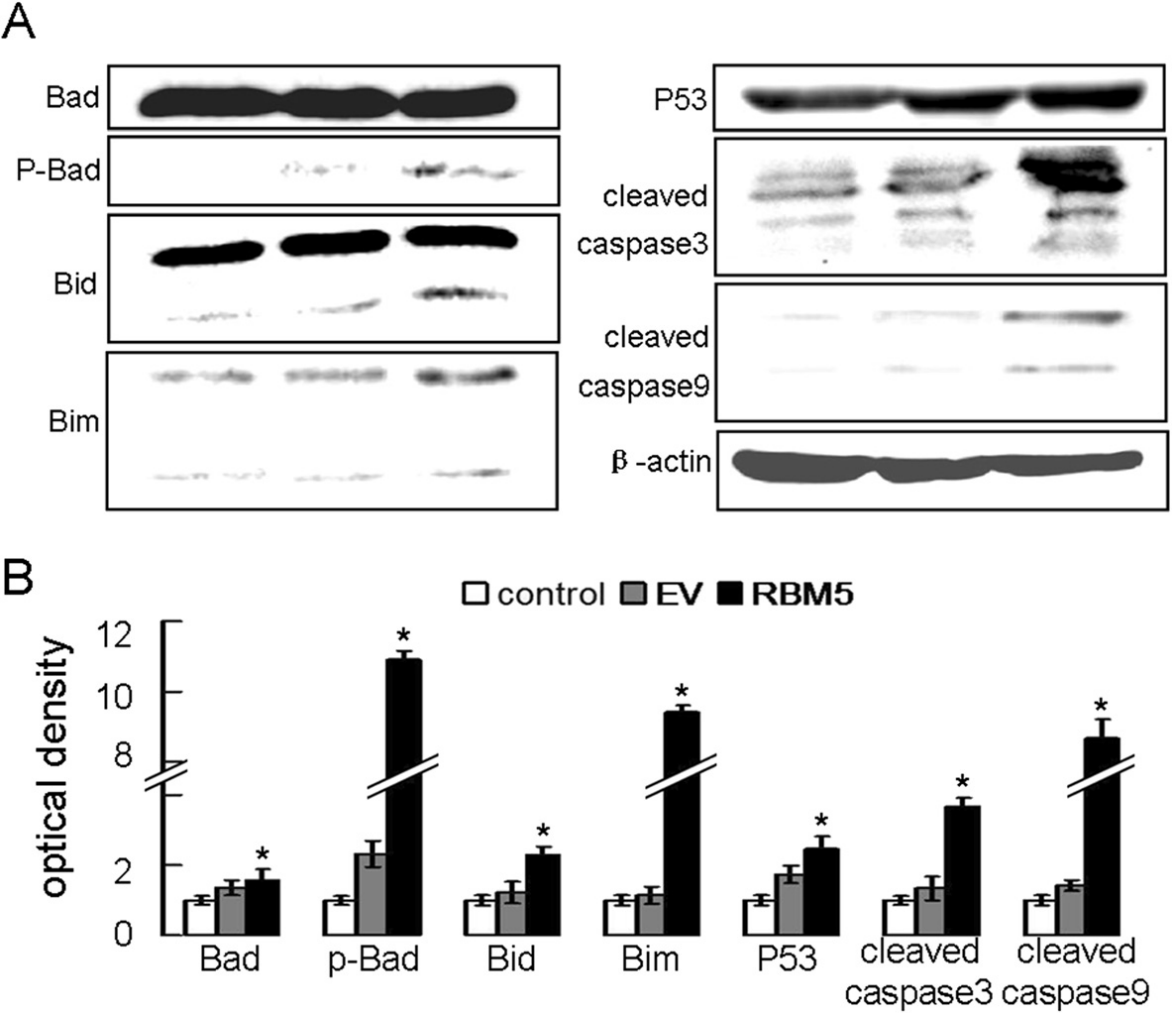
**RBM5 induces apoptosis-related genes expression.** Cellular proteins were extracted from PC-3 cells transfected with pcDNA3.1 or pcDNA3.1.1-RBM5 plasmids for 48 h. (**A**) Expression of Bad, p-Bad, Bid, Bim, P53, cleaved-caspase3, and cleaved-caspase9 were determined by western blot. (**B**) Quantitative data from A by densitometry using Quantity One software (Bio-Rad). * indicates significant difference as compared to the control and EV groups (*P* <0.05).

To investigate potential molecular mechanisms underlying RBM5-induced cell apoptosis, we next examined the expression of some apoptosis-related genes including P53, Bid, Bim, Bad, p-Bad, cleaved-caspase3, and cleaved-caspase9. We observed that the expression of P53, Bim, Bid, p-Bad, cleaved-caspase3, and cleaved-caspase9 proteins was increased significantly when RBM5 was overexpressed compared to that in the control cells (Figure 5). RBM5 might play an import role in the mitochondrial apoptotic pathway.

## Discussion

RBM5 is one of nine genes downregulated in metastasis and was identified as one of the 17-gene signatures associated with metastasis and poor clinical outcomes in various solid tumor types [22]. RBM5 gene encodes a number of alternative RNA splice variants with differing abilities to enhance, sensitize, or suppress apoptosis [18,23-25]. Other findings show that RBM5-knockdown upregulated the expression of genes involved in cell adhesion, migration, and motility [26]. RBM5 mRNA was downregulated in spontaneously developing human tumors such as human schwannomas [9] as well as in transformed cells, such as ras-transformed Rat-1 rat embryonic fibroblastic cells [8]. All of these findings suggest that patients with lower levels of RBM5 have a poor prognosis, possibly due to tumor progression, metastasis, and resistance to cancer therapy. In the preliminary study, our group validated that the expression of RBM5 is lower in human lung cancer tissues than normal tissues did [11], the overexpression of RBM5 inhibit the proliferation and induce the apoptosis of human lung cancer A549 cells [15]. But whether the expression levels of RBM5 have changes in prostate cancer had not been reported. In this study, we first examined RBM5 protein expression in prostate cancer tissue and normal prostate tissue with immunohistochemistry staining. The results indicated that prostate cancer tissue expressed lower RBM5 levels than the normal prostate tissue. This implies a correlation between prostate cancer and the expression of RBM5 and further suggests that RBM5 plays a critical role in the occurrence and development in prostate cancer.

In order to explore the role of RBM5 in prostate cancer, we transfected pcDNA3.1-RBM5 vector into human prostate cancer PC-3 cells, and confirmed the overexpression of RBM5 RNA and protein by RT-PCR and western blot. We further observed the changes in PC-3 cells with the RBM5 overexpression, the results showed that RBM5 overexpression significantly inhibited cell proliferation and induced apoptosis in PC-3 cells. The expression of cleaved caspase3 and cleaved caspase9 was upregulated in pcDNA3.1-RBM5 transfected cells. These results were similar to our previous findings on lung cancer and provided evidence that RBM5 might function as a tumor suppressor and apoptosis inducer for prostate cancer.

Thus, it is likely that the ability to modulate apoptosis is the central to the putative tumor suppressor activity of RBM5. The increasing evidence showing the potential molecular mechanism of tumor suppressive activity of RBM5 involves both cell cycle arrest and apoptosis. Tobayashi *et al.* showed that RBM5 contributes to p53 transcriptional activity after DNA damage and that growth suppression and apoptosis mediated by RBM5 are linked to activity of the tumor suppressor protein P53 [27]. By western blot, we observed a significantly increased expression of wild-type P53, just similar to the results of Tobayashi *et al*. Oh *et al.* showed that the expression of Bax protein was increased in RBM5-transfected A549 cells [14]. Bax is a cytosolic monomer in healthy cells, but it changes conformation during apoptosis, integrates into the outer mitochondrial membrane and oligomerizes [28]. How the homo-oligomers form is unclear. It was reported that the Bax-like proteins could assume both a ‘BH3 donor’ and a ‘BH3 acceptor’ conformer within the membrane environment. The BH3-only proteins are sentinels that detect developmental death cues or intracellular damage. In healthy cells, they are restrained in diverse ways, including sequestration on the cytoskeleton. When unleashed by death signals, they switch off survival function by inserting their BH3 domain into a groove on their pro-survival relatives [29]. In this study, we detected the member of BH3-only proteins Bid, Bim, Bad, and p-Bad. The result showed that the expression of cleaved Bid, Bim, and p-Bad were increased in RBM5-transfected A549 cells. We demonstrated that BH3-only proteins play critical roles in RBM5-induced apoptosis.

BH3-only proteins can be activated by transcriptional induction, by post-translational modification or by liberation from endogenous inhibitors. They can exert their pro-apoptotic action by different mechanisms. Individual BH3-only proteins are normally held in check by diverse mechanisms, and might transducer specific death signals [30]. Bad preferentially interact with anti-apoptotic Bcl-2 and Bcl-XL, dissociating them from BH3 or Bax/Bak-like proteins, which in turn mediate MMP [31]. Bid is relatively inactive until proteolytically cleaved. Bid seems to promote death by activating Bax and Bak to oligomerize and form pores in the membrane, and it might also inactivate pro-survival relatives [32]. Bim is sequestered by binding to dynein light chains that are associated with the microtubules [33]. As Bim does not bind to Bax or Bak, it must act by preventing the pro-survival proteins from inhibiting the activation of Bax and Bak. Along with the study of Oh’s, this study indicated that the overexpression of RBM5 might induce expression of the BH3-only proteins Bim, p-Bad and cleaved Bid, BH3-only proteins further engage directly and activate Bax, this led to changes in the mitochondrial membrane potential, cytochrome c release into the cytosol, and enhanced caspase-9 and caspase-3 activities, thereby inducing apoptosis.

## Conclusions

In summary, we have shown that RBM5 has a correlation with prostate cancer. The ectopic introduction of RBM5 can induce the apoptosis of human prostate cancer PC-3 cells by modulating the mitochondrial apoptotic pathway, and thus RBM5 may be a promising target for gene therapy on prostate cancer.

## Abbreviations

MTT: 3-(4,5-dimethylthia-zol-2-yl)-2,5- diphenyltetrazolium bromide; PCa: Prostate cancer; RBM5: RNA Binding Motif 5.

## Competing interests

The authors declare that they have no competing interests.

## Authors’ contributions

LZ performed all the experiments and drafted the manuscript. RL collected and provided the tissues. CS, PL, and JL have contributed part of the experiment, research design, and the data collection. KW oversaw the design of the study, was involved in the critically revised the manuscript. All authors have read and approved the final version of the manuscript.
